# Analysis of Enamel Loss by Prophylaxis and Etching Treatment in Human Tooth Using Optical Coherence Tomography: An *In Vitro* Study

**DOI:** 10.1155/2019/8973825

**Published:** 2019-03-06

**Authors:** Naresh Kumar Ravichandran, Hemanth Tumkur Lakshmikantha, Hyo-sang Park, Mansik Jeon, Jeehyun Kim

**Affiliations:** ^1^School of Electronics Engineering, College of IT Engineering, Kyungpook National University, Daegu 41566, Republic of Korea; ^2^Department of Orthodontics, School of Dentistry, Kyungpook National University, Daegu 41940, Republic of Korea

## Abstract

Bonding of braces is an essential part in contemporary orthodontic treatment. For the proper strength of bracket bonding, enamel conditioning or surface treatment on tooth surface is required. Treatment on the tooth surface such as prophylaxis smoothing with pumice and enamel etching results in considerable damages to the enamel surface of the tooth. In this study, we have proposed optical coherence tomography as a noninvasive imaging technique for the evaluation of damage induced during such treatment procedures. Using depth intensity analysis of the obtained cross-sectional images, the damage resulting to the enamel surface was studied after prophylaxis smoothening and etching steps.

## 1. Introduction

Use of fixed braces during orthodontic treatment procedures plays a key role in tooth movement. Bonding of brackets greatly varies from individual orthodontic practitioners. Similarly, the steps involved before bracket bonding can also vary depending on the treatment and necessity depending on the practitioners [1].

In clinical practice, in most cases, prophylaxis removing debris, smoothing of the tooth surface, and enamel etching are most commonly used prerequisite steps followed before bonding of brackets on the tooth [2–5]. The purpose of etching is the partial dissolution of the enamel minerals that help in mechanical retaining between the orthodontic resin tag and tissue pores. This process creates a significant roughness on the tooth surface which plays a major role in the stability of braces attachment. This is influenced by the etching agent composition and the application time of the etching on the tooth surface. Furthermore, due to the effect of dissolving enamel, it is of crucial importance to perform the procedure cautiously and skillfully to obtain best results. Similarly, applying prophylaxis paste and prophy of the enamel surface with abrasives leads to slight erosion and abrasion of the enamel surface. The influencing factors on surface roughness during this process mainly depend on the composition, application of force, and application time of the solution. Earlier studies on different etching and prophy methods have stated the optimal application time, and the use of abrasive grades from coarse to fine has been published [2, 6, 7]. Also, using destructive and nondestructive imaging methods for measurement of enamel loss and surface roughness by these methods have been published earlier.

It would be of greater advantage if practitioners can employ a noninvasive and cross-sectional measurement tool for assessment of tooth while performing the treatment procedures. Optical coherence tomography (OCT) is one such method which offers noncontact, cross-sectional images of samples with micrometer resolution in real time. Many previous studies on the application of OCT in dental experiments and orthodontic studies have shown the emerging capability of using OCT as a reliable technique for noninvasive, cross-sectional imaging tool for such procedures [8–20]. In this study, we have shown the potential capability of OCT for enamel loss measurement during orthodontic prophy pumice and enamel etching procedures.

## 2. Materials and Methods

### 2.1. Specimen Preparation

All the teeth samples were obtained from the Department of Orthodontics, School of Dentistry, Kyungpook National University, Daegu, Republic of Korea. All the experiments were performed in accordance with the guidelines of the Institutional Animal and Human Care and Use Committee of Kyungpook National University (no. 2017-0145-1). The collected samples were premolars, extracted for orthodontic and periodontal reasons which belonged to patients who were within the age group of 20–30 years. The collected tooth samples were inspected individually and selected based on the uniformity of the sample. Once the teeth samples were selected, the samples were debrided of any soft tissues and stored in a solution of demineralized water and crystal thymol (0.1%) at room temperature and were used in the experiment within 2 weeks of extraction. The samples were not cleaned with an abrasive material, so as to prevent any surface damage to the enamel surface. The tooth surface was cleaned with fluoride-free toothpaste with gentle pressure. Following the samples were rinsed gently with distilled water and dried with compressed air. The study was carried out with a total of 30 teeth samples, which was divided into three groups of 10 teeth samples in each group. Once the tooth samples were selected, they were mounted onto a wax brick in order to maintain stability of the samples. The mounted samples were then prepared for the experiment. An applicator tip, commonly used in restorative dentistry was used to carry the chemicals and to apply them onto the selected area of the tooth in order to prevent spilling of the chemicals to the control surface. In samples, wherein spillage was suspected, the samples were discarded and are replaced. The first group, 10 teeth samples, received a thorough prophylactic treatment with plain pumice for 10 seconds. In the second group, teeth samples were treated with 37% orthophosphoric acid for 15 seconds. In the third group, the samples were first prophylactic treated with plain pumice for 10 seconds, followed by 37% orthophosphoric acid for 15 seconds. Furthermore, in conventional orthodontics, brackets are attached on the buccal surface of the tooth. In order to replicate the conditions as it is in the oral cavity, in our experiment, we used the buccal/facial surface of the tooth for experimental purposes in all the experimental groups. Additionally, the buccal surface of the tooth was further divided into right and left surface. The experiment was conducted on the right side, and the left side surface was treated as a control surface on all samples in all the groups.

### 2.2. Optical Coherence Tomography System Specification

**Figure 1:**
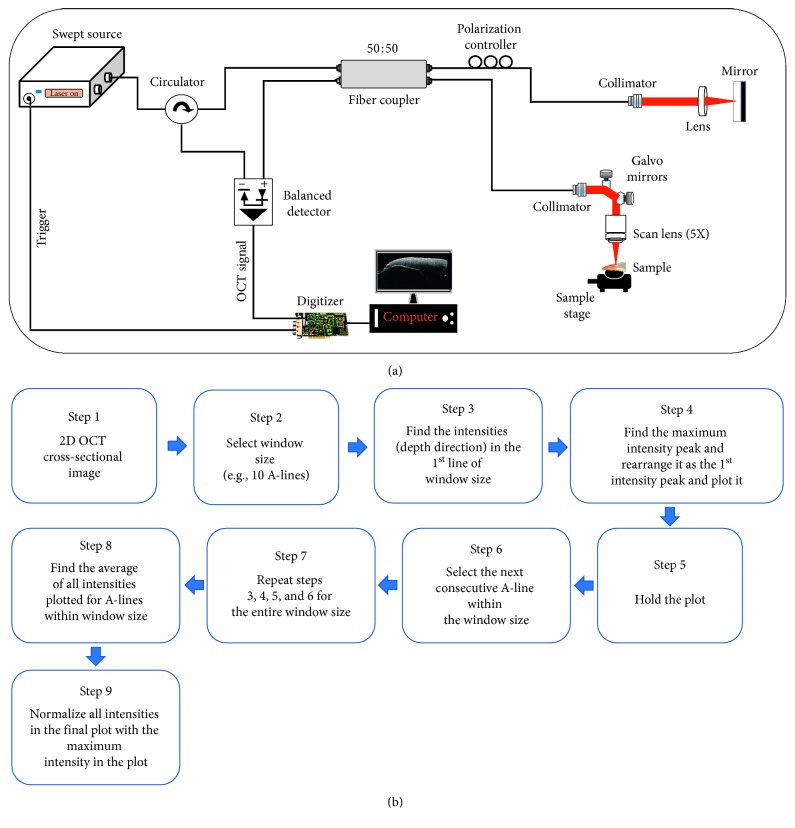
(a) The schematic diagram representing the hardware setup of the SS-OCT system. (b) The flow diagram showing the steps in depth intensity analysis algorithm.

A commercially available swept source optical coherence tomography (SS-OCT) system (OCS1310V1, Thorlabs, New Jersey, United States) was used for imaging and measurement. The schematic representation of the SS-OCT system is shown in Figure 1(a). The Thorlabs SS-OCT system was powered by a microelectromechanical system (MEMS) tunable vertical-cavity surface-emitting laser (VCSEL) swept laser source which was centered at 1310 nm. The sweeping rate was 100 kHz, and the full width at half maximum (FWHM) spectral bandwidth of the source was 97 nm. A small portion of the output beam from the VCSEL cavity module was launched into a Mach–Zehnder interferometer (MZI) clock module to achieve real-time optical clocking, upon which OCT spectral fringes can be evenly sampled in wavenumbers with the referenced MZI signals, eliminating the necessity of postprocessing for fringe resampling. The remaining output beam of the laser source was coupled into the main OCT interferometer, which is connected to a circulator. The output arm of the circulator (within the interferometer) is connected to an optical coupler of ratio 50 : 50. The coupler outlets are connected to a sample arm and reference arm setup. The back-reflected optical signals interfere in the optical coupler and the output end of the coupler and the final output from the circulator arm are connected to the positive and negative terminals of the dual-balanced photodetector. The sample arm setup consists of a 2D galvanometer scanner and an objective scan lens (LSM03, Thorlabs, New Jersey, United States) of NA = 0.055. The reference arm setup comprised of a collimator, a lens, and a highly reflective mirror. The interference signal from the dual-balanced photodetector is then linearly sampled by a 12 bit, 500 MS/s data acquisition card. Thus, the depth-dependent reflectivity profile (A-line) was produced by fast Fourier transformation of the sampled fringe signals, and by using the galvanometer scanner, a two-dimensional cross-sectional OCT image is generated in real time. The sensitivity of the OCT system is 105 dB. The axial and transverse resolutions of the system (in air) were 18 *µ*m and 25 *µ*m, respectively. The acquired B-scan (2D image) data after processing consist of 2000 A-scans (depth scans) with each A-scan consisting of 1407 pixels. The three-dimensional image (C-scan) was formed by 500 successive B-scans. The axial and transverse resolutions measured in the air are 16 and 25 *μ*m, respectively. The scan range of one 2D cross-sectional image (B-scan) was set to 5 mm, and the volumetric scan range (C-scan) was set to 5 × 5 mm.

### 2.3. Depth Intensity Analysis Algorithm

For analyzing the obtained OCT images, a MATLAB-based software program was developed and utilized for intensity peak detection in depth direction of the cross-sectional 2D OCT images. The flow diagram of the algorithm is shown in Figure 1(b). The program searches for intensity peaks in the OCT images which are given within the desired window size. We used a window size of 10 for all the samples. Following this, the algorithm sequentially detects for maximum intensity of the A-scan (depth scan) signals. After which, the program rearranges of all the detected peak positions of A-scans within the window size while matching peak intensity index in the A-scans to flatten the image. The index positions respective to high intensity are rearranged and matched linearly to get a flattened plot. The rearrangement is executed in such a way that the first intensity peaks were retrieved from every A-scan of a 2D image and plotted at the beginning of the A-scan plot. It is to be noted that the absence of intensity peaks in the plot may be due to the presence of layers which are smaller than the maximum resolution of the system. Finally, all the rearranged and flattened A-scan lines were summed up and averaged, so as to obtain an averaged depth intensity profile. Then, the obtained depth intensity profiles were divided by the maximum value to obtain a normalized depth intensity plot for the desired window size of the 2D OCT image.

## 3. Results and Discussion

### 3.1. 2D and 3D OCT Image Analysis

The scan range was set to 5 × 5 mm while scanning all the teeth samples. To better visualize the internal structures, the 3D volumetric dataset was viewed with the help of volume rendering software. Then, different section planes in sagittal, coronal, and transverse directions were used for identification of individual layers and image analysis.

**Figure 2:**
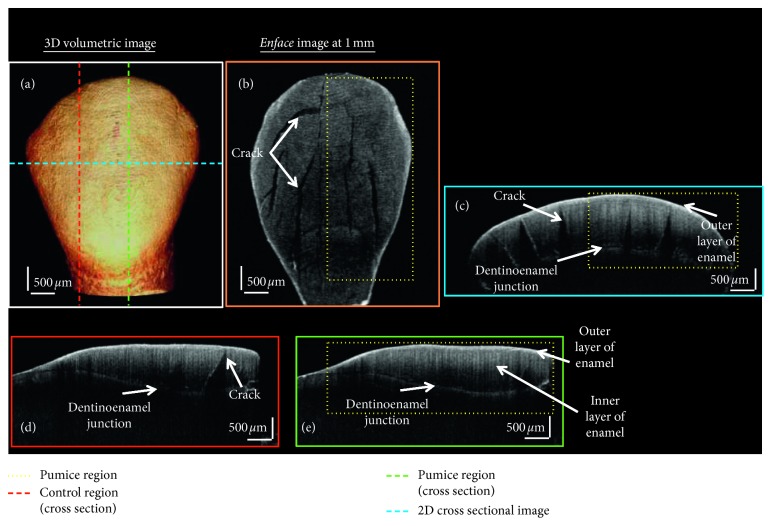
OCT images of prophylactically treated tooth sample. (a) 3D volumetric OCT image. (b) The *en face* image at 1 mm. (c) A 2D cross-sectional OCT image. (d) Sagittal plane showing the 2D cross-sectional image in the control region. (e) Sagittal plane showing the 2D cross-sectional image in the prophylactically treated region.

Figure 2 shows a representative tooth that was scanned using SS-OCT of only prophylactic with a plain pumice treated group. Figure 2(a) is the 3D volumetric image of the tooth. Figure 2(b) is the *en face* (transverse plane) image which was obtained from the volumetric dataset at 1 mm depth below the top surface of the enamel. Figure 2(c) is the 2D cross-sectional (coronal plane) SS-OCT image in the coronal plane. And Figures 2(d) and 2(e) are the cross-sectional OCT image in the sagittal plane. The blue, red, and green dashed lines shown in Figure 2(a) correspond to the coronal and sagittal plane as shown in 2(c)–2(e).  2(d) is the control region, and 2(e) is the prophylactically treated region. The yellow-dotted region in 2(b), 2(c), and 2(e) is the prophylactically treated region. Figures 2(b)–2(d) shows presence of few microcracks in the 2D OCT images. It is to be noted that the samples were carefully selected by an expert orthodontist during the selection of tooth samples. These were microcracks which were not predominantly visible during visual inspection. Even though it is true that ideal conditions for experiments would be tooth without any microcracks or microdamage, the presence of the cracks in the enamel structure can lead to the variations in total presence of enamel constituents in the tooth structure. But, in clinical practice, it is possible for the occurrences of microcracks and microdamage on tooth surfaces where the orthodontic procedures are carried out. Hence, to evaluate the experiment in circumstances that is similar to clinical procedures, we did not discard the teeth that contained microcracks or microdamage. Furthermore, all the experimental groups had maximum of 1 to 2 samples which contained such deformities in the enamel structure. This tooth sample is included as one of the representative images of the pumice treated group to show the changes in the internal structural anatomy of the tooth surface in the presence of microcracks in the enamel region. The presence of microcracks in the control and treated region indicates that the microcracks were present prior to treatment procedures. Additionally, it can be observed that the treatment procedure did not lead to any increase or changes to the microcrack or to its surrounding region in the enamel surface.

**Figure 3:**
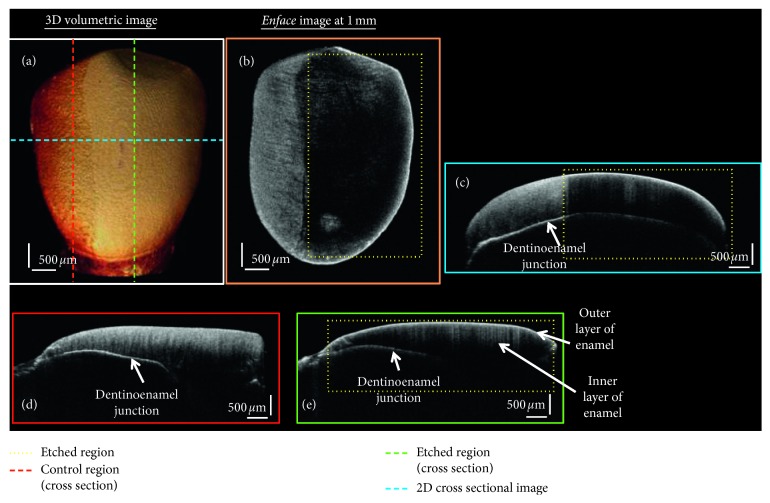
OCT images of the 37% orthophosphoric etch treated tooth sample. (a) 3D volumetric OCT image. (b) The *en face* image at 1 mm. (c) A 2D cross-sectional OCT image. (d) Sagittal plane showing the 2D cross-sectional image in the control region. (e) Sagittal plane showing the 2D cross-sectional image in the etch treated region.

Figure 3 shows the OCT images of a representative tooth sample of the 37% orthophosphoric etch treated group. Figure 3(a) is the volumetric image, and adjacent to it is the *en face* image at 1 mm depth from the tooth surface. The two-dimensional cross-sectional OCT image is shown in 3(c) that is obtained from the position which is shown in the dashed blue line, as shown in Figure 3(a). Figures 3(d) and 3(e) are the cross-sectional OCT image in the sagittal plane at nonetched and etched regions of the tooth, respectively. The position of these obtained images is shown as red- and green-dashed line in the 3D OCT image (Figure 3(a)). It can be observed that the absence of intensity in the cross-sectional and *en face* images corresponds to the enamel loss in the tooth due to etching. These etched regions are shown as yellow-dotted regions in the OCT images.

**Figure 4:**
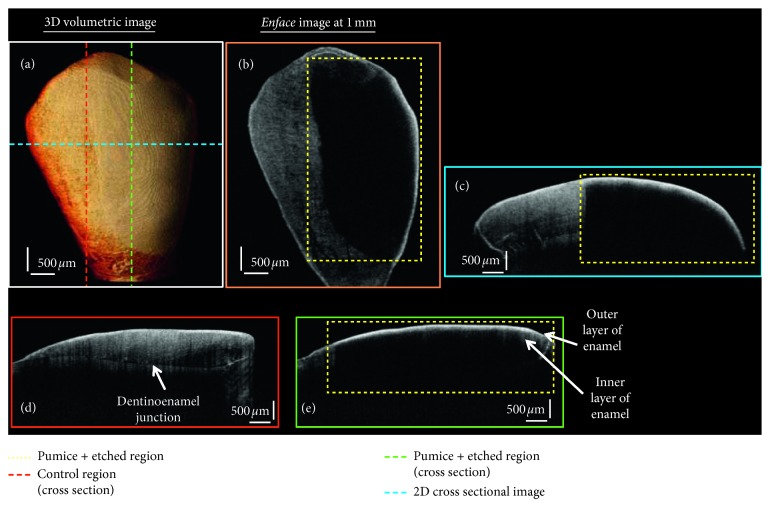
OCT images of the prophylactic and etch treated tooth sample. (a) 3D volumetric OCT image. (b) The *en face* image at 1 mm. (c) A 2D cross-sectional OCT image. (d) Sagittal plane showing the 2D cross-sectional image in the control region. (e) Sagittal plane showing the 2D cross-sectional image in the pumice and etch treated region.

Figure 4 shows the OCT images of a representative tooth sample of the group that was treated with prophylactic with plain pumice for 10 seconds, followed by 37% orthophosphoric etching for 15 seconds. Figure 4(a) is the volumetric image, and adjacent to it is the *en face* image at 1 mm depth from the tooth surface. Figure 4(c) is the 2D OCT cross-sectional image, and its corresponding position is shown as a dashed-blue line in Figure 4(a). Figures 4(d) and 4(e) are the cross-sectional OCT image in the sagittal plane at the nontreated and treated regions of the tooth, respectively. The position of these obtained images is shown as red- and green-dashed line in the 3D OCT image (Figure 4(a)). It can be observed that the absence of intensity in the cross-sectional and *en face* images corresponds to the enamel loss in the tooth due to etching. These etched regions are shown as yellow-dotted regions in the OCT images. The internal structures of the tooth in the 2D cross-sectional OCT images (shown in Figures 3 and 4) were identified by correlating the OCT images with its respective depth intensity profile plots.

### 3.2. Depth Intensity Analysis of OCT Images

**Figure 5:**
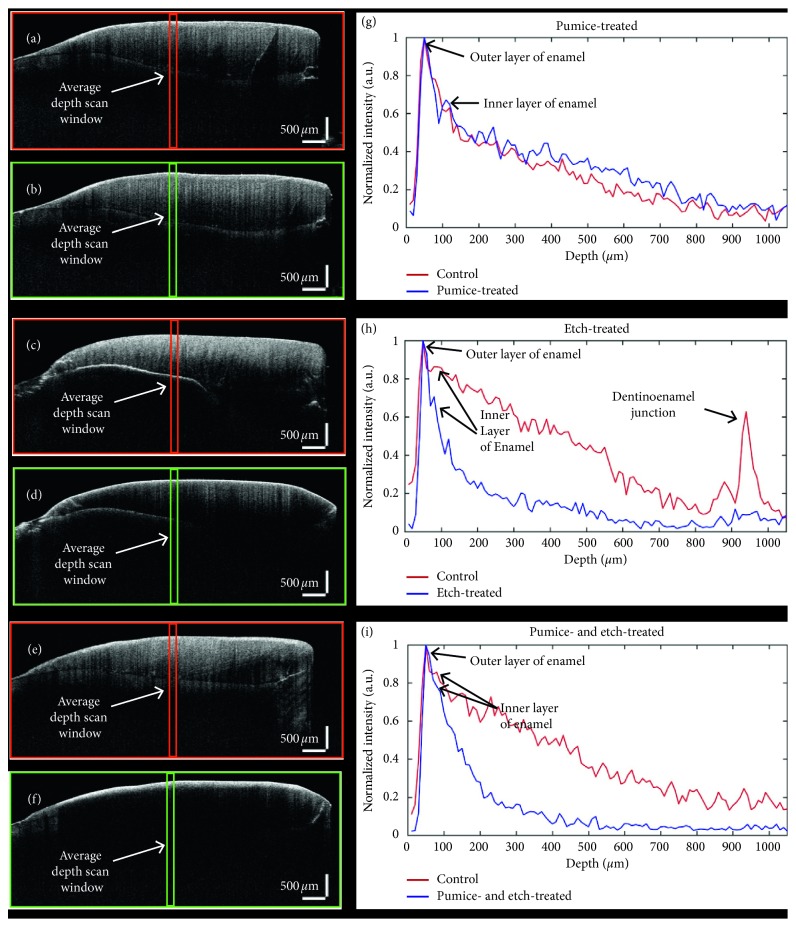
OCT cross-sectional images and the corresponding depth intensity plots of all three groups. (a, b, and g) The OCT images of prophylactically treated tooth sample and its respective depth intensity plots. (c, d, and h) The OCT images of the 37% orthophosphoric etch treated tooth sample and its respective depth intensity plots. (e, f, and i) The OCT images prophylactic and etch treated tooth sample and its respective depth intensity plots.

As previously mentioned in Section 2.3, the obtained OCT images underwent depth intensity analysis by the developed software algorithm. The 2D cross-sectional images control and its corresponding treated regions of a sample for all the three groups were used for depth intensity analysis. The averaging window size was 10, and the averaged window regions in the OCT images are shown with red and green box regions, as shown in Figure 5. The averaged depth intensity plotting was evaluated with various values of window size ranging from 1 to 15; it was observed that the window size of 10 was most optimal, as it had least noise without changes in the width of the intensity peak. To get a corelatable result, the averaged and normalized depth intensity profiling was performed in the center position of the 2D cross-sectional OCT images. This was helpful in maintaining the position (aligning) the analyzed position in all the 2D OCT images. Figures 5(a) and 5(b) are only prophylactic with plain pumice treated sample images of the control and treated region, respectively, and 5(g) is its depth intensity graph.  In 5(g), the red plot indicates the control region and blue plot indicates the treated region. In this study, we used the left part of the tooth as the control region for all samples. This was done since the enamel structural constituency varies from one sample to another. To get a comparable and corelatable analysis and measurements between the control and treated region, the left part of all tooth samples was used as the control region. Additionally, to compensate for this variation, we used a sample number of 10 in each group. Furthermore, the intensities in plots were normalized with the highest peak intensity in each plot. This ensured to perform comparative analysis during enamel measurement without any errors. From the obtained depth intensity plots, it could be seen that there is a very little deviation from the treated graph in depth intensity fluctuation.

This could have been due to internal structural differences in the scanned position of the sample. Also, prophylaxis is used for surface roughening and stain removal. And as per expectation, not much of enamel loss can be seen in this procedure. On the other hand, in 37% orthophosphoric etched sample, the variation is much higher. Figures 5(c) and 5(d) are the cross-sectional images of the control and etch treated regions on a tooth. And in its respective plots in 5(h), the depth intensity of the control region (red plot) is much higher than the treated etch treated region (blue plot). This difference is due to the loss of mineral in the enamel surface. The intensity peak between 900 and 1000 in *x*-axis in Figure 5(h), denotes the dentinoenamel junction in tooth. The reason it is not visible in other plots is due to the fact that the backscattering intensity decreases as the depth increases in OCT images. This could be observed as the brightness of intensity, and continuity of the dentinoenamel junction declines as increase in depth of 2D cross-sectional OCT images. Even though the presence of dentinoenamel junction in the tooth may be continuous, due to penetration limitations in OCT images, this layer is not so clearly visible in some 2D OCT images. Figures 5(e) and 5(f) are the cross-sectional OCT images of a tooth which was first treated with prophylactic with plain pumice for 10 seconds and followed by 37% orthophosphoric etching for 15 seconds. Figure 5(i) is the respective depth intensity plot of 5(e) and 5(f) that has been highlighted with red and green box regions. Comparable to the only etch treated samples depth intensity plots, the pumice and etch treated plot also shows very high intensity differences. But the internal layers like the enamel-dentin junction are barely visible in this group. The process of etching in the tooth surface leads to demineralization of enamel in tooth. This leads to increase in porosity in tooth; this can aid in more water deposition on the surface. This leads to reduction of reflection of the laser source from the sample surface. Furthermore, the demineralization by the etching process results in higher backscattering of laser light from the uppermost surface of the tooth samples which may be a resultant due to structural changes in the tooth surface.

The calculated enamel thickness values after each treatment procedures for all 30 samples (10 samples of each group) have been given in Table 1. The enamel thickness in the treated and control areas of samples was obtained from the averaged and normalized depth intensity plots (averaged and normalized A-scan analysis). The representative plots are shown in Figures 5(g)–5(i). In these plots, the first and the second intensity peak represents the outer and the inner enamel thickness of the imaged tooth surface. It is to be noted that all the measured enamel thickness was analyzed for both enamel (outer and inner) structures. The orthophosphoric acid treated samples showed high reflectivity from the outer enamel surface, which might have been resultant of the changes in structure, refractive index, and reflectivity of the tooth surface. This can be seen in the 37% orthophosphoric etch treated samples, and also it further increases in the samples that were treated by both pumice and 37% orthophosphoric etch treated samples. The overall composition of the enamel structure was measured by taking the total width of the intensity peaks which were at least 30 percent or higher (intensity of 0.3 A.U. or higher in the plots) of the overall continuous intensity peaks in the depth profile plots. From the calculated values, it can be observed that the enamel thickness after pumice treatment is not much changed. There was, however, reduced enamel thickness of samples after only etch and pumice-etch treatment with the least enamel thickness in pumice-etch treatment samples. And it is to be noted that only very little difference in enamel thickness was observed between only the etch treated sample group and the etch plus pumice treated sample group. The complete set of calculated enamel thickness values of all 30 samples is given in the supplementary materials (available here).

It is a globally acknowledged fact that adequate bond strength is necessary for successful orthodontic treatment. Successful etching is necessary for strong bond between teeth and bracket. Conventionally, pumice was used before etching. But many clinicians do not like because of additional time constraints in an extrastep before etching. Using OCT, we wanted to check enamel loss with pumice and etch combination to check that hypothesis. In our study, we devised three experimental groups of 10 human tooth samples in each group. First group of samples received a thorough prophylactic treatment with plain pumice for 10 seconds. Second group of samples was treated with 37% orthophosphoric acid for 15 seconds. In the third group, the samples were first prophylactic treated with plain pumice for 10 seconds, followed by 37% orthophosphoric acid for 15 seconds.

In the pumice only treated group, we observed the amount of enamel distortion is not adequate to form successful resin tags. An additional step of chemical is necessary to increase surface area that is necessary to create adequate resin tags that is necessary for a strong bond between teeth and bracket. On the other hand, the enamel loss is minimal. It should also be mentioned, that when pumice is used, microscopically, there might be remnants of the pumice material left behind, which can hamper bond strength of the bracket to the youth material. In the second group of samples, only etching is done. This is the most common procedure used in clinical practice. In this sample group, we observed the enamel loss is mainly due to leeching of the acid into the enamel and resultant demineralization of the enamel structure. This is important for creation of resin tags and bond strength of bracket to the tooth surface. The third group consisted of samples which underwent both pumice and etch and as a resultant, showed increased enamel loss. This is due to the cumulative effect of pumice and etch. It can be said that the resultant bond strength could be higher in this group but at the cost of increased enamel loss. Also due to the pumice prophylaxis, remnants of pumice can accumulate at the porosities and cause weaker bonds. In case of bracket loss, repeated producers of pumice and etch accumulate the enamel loss, and at the end of treatment, patient might be susceptible to dentinal sensitivity.

## 4. Conclusion

Through this study, we have shown the potential capability of using optical coherence tomography (OCT) as a noninvasive, cross-sectional imaging tool that can be used for enamel loss assessment during orthodontic treatment procedures such as prophylaxis and enamel etching. With the help of OCT, we have shown the internal structural changes that occur during prophylaxis and etching treatment procedures. Also, with the help of depth intensity analysis, the enamel during these procedures was studied and reported. In the future, with advancements, OCT technologies such as system portability, improved resolution, higher depth-resolved images, and compact handheld scanning probes, OCT system can greatly assist orthodontic and dental practitioners in clinical practices.

## Figures and Tables

**Table 1:** Calculated enamel thickness (*µ*m) after each treatment procedure.

Treatment	Control				Treated			
	Min.	Max.	Mean	Standard deviation	Min.	Max.	Mean	Standard deviation
Pumice	471.07	554.67	517.51	28.46	476.15	550.32	521.19	23.05
Etch	482.05	577.34	532.13	34.77	140.50	171.44	156.48	10.58
Pumice and etch	488.76	575.25	527.75	24.12	142.72	165.72	151.23	8.72

## Data Availability

The JPG data used to support the findings of this study are included within the article.

## Supplementary Materials

Supplementary Materials The complete set of calculated enamel thickness values of all 30 samples is given in the supplementary materials. Supplementary 1 includes the calculated enamel thickness values measured in the control region of all 30 samples. Likewise, supplementary 2 includes the calculated enamel thickness values measured in the treated region of all three groups: pumice treated, etch treated, and pumice plus etch treated (total of 30 samples).
