# Supplementary material for: Analysis of Enamel Loss by Prophylaxis and Etching Treatment in Human Tooth Using Optical Coherence Tomography: An In Vitro Study
Source: J Healthc Eng. 2019 Mar 6;2019:8973825. doi: 10.1155/2019/8973825 (PMC6431396; doi:10.1155/2019/8973825)
Supplement: Supplementary Materials — The complete set of calculated enamel thickness values of all 30 samples is given in the supplementary materials. Supplementary 1 includes the calculated enamel thickness values measured in the control region of all 30 samples. Likewise, supplementary 2 includes the calculated enamel thickness values measured in the treated region of all three groups: pumice treated, etch treated, and pumice plus etch treated (total of 30 samples). [file 8973825.f1.zip › 8973825.f1/Supplementary1_JHE_2670850.docx]

| **Control** | | | | | | |
| --- | --- | --- | --- | --- | --- | --- |
|  | **Sample No** | **Enamel Thickness** | **Min** | **Max** | **Mean** | **Standard deviation** |
| **Pumice** | 1.00 | 543.65 | 471.07 | 554.67 | 517.51 | 28.46 |
|  | 2.00 | 501.68 |  |  |  |  |
|  | 3.00 | 492.49 |  |  |  |  |
|  | 4.00 | 545.52 |  |  |  |  |
|  | 5.00 | 544.11 |  |  |  |  |
|  | 6.00 | 471.07 |  |  |  |  |
|  | 7.00 | 507.19 |  |  |  |  |
|  | 8.00 | 554.67 |  |  |  |  |
|  | 9.00 | 521.23 |  |  |  |  |
|  | 10.00 | 493.49 |  |  |  |  |
| **Etch** | 1.00 | 505.19 | 482.05 | 577.34 | 532.13 | 34.77 |
|  | 2.00 | 482.05 |  |  |  |  |
|  | 3.00 | 505.27 |  |  |  |  |
|  | 4.00 | 568.93 |  |  |  |  |
|  | 5.00 | 577.34 |  |  |  |  |
|  | 6.00 | 533.99 |  |  |  |  |
|  | 7.00 | 490.43 |  |  |  |  |
|  | 8.00 | 567.91 |  |  |  |  |
|  | 9.00 | 554.30 |  |  |  |  |
|  | 10.00 | 535.86 |  |  |  |  |
| **Pumice**  **and Etch** | 1.00 | 508.26 | 488.76 | 575.25 | 527.75 | 24.12 |
|  | 2.00 | 575.25 |  |  |  |  |
|  | 3.00 | 542.72 |  |  |  |  |
|  | 4.00 | 546.16 |  |  |  |  |
|  | 5.00 | 525.44 |  |  |  |  |
|  | 6.00 | 488.76 |  |  |  |  |
|  | 7.00 | 513.10 |  |  |  |  |
|  | 8.00 | 539.39 |  |  |  |  |
|  | 9.00 | 516.41 |  |  |  |  |
|  | 10.00 | 522.05 |  |  |  |  |
