# Supplementary material for: Analysis of Enamel Loss by Prophylaxis and Etching Treatment in Human Tooth Using Optical Coherence Tomography: An In Vitro Study
Source: J Healthc Eng. 2019 Mar 6;2019:8973825. doi: 10.1155/2019/8973825 (PMC6431396; doi:10.1155/2019/8973825)
Supplement: Supplementary Materials — The complete set of calculated enamel thickness values of all 30 samples is given in the supplementary materials. Supplementary 1 includes the calculated enamel thickness values measured in the control region of all 30 samples. Likewise, supplementary 2 includes the calculated enamel thickness values measured in the treated region of all three groups: pumice treated, etch treated, and pumice plus etch treated (total of 30 samples). [file 8973825.f1.zip › 8973825.f1/Supplementary2_JHE_2670851.docx]

| **Treated** | | | | | | |
| --- | --- | --- | --- | --- | --- | --- |
|  | **Sample No** | **Enamel Thickness** | **Min** | **Max** | **Mean** | **Standard deviation** |
| **Pumice** | 1.00 | 527.69 | 476.15 | 550.32 | 521.19 | 23.05 |
|  | 2.00 | 515.73 |  |  |  |  |
|  | 3.00 | 476.15 |  |  |  |  |
|  | 4.00 | 534.35 |  |  |  |  |
|  | 5.00 | 550.32 |  |  |  |  |
|  | 6.00 | 524.56 |  |  |  |  |
|  | 7.00 | 496.52 |  |  |  |  |
|  | 8.00 | 543.24 |  |  |  |  |
|  | 9.00 | 538.30 |  |  |  |  |
|  | 10.00 | 505.01 |  |  |  |  |
|  | | | | | | |
| **Etch** | 1.00 | 153.02 | 140.50 | 171.44 | 156.48 | 10.58 |
|  | 2.00 | 167.66 |  |  |  |  |
|  | 3.00 | 149.65 |  |  |  |  |
|  | 4.00 | 152.25 |  |  |  |  |
|  | 5.00 | 163.66 |  |  |  |  |
|  | 6.00 | 148.25 |  |  |  |  |
|  | 7.00 | 171.44 |  |  |  |  |
|  | 8.00 | 149.34 |  |  |  |  |
|  | 9.00 | 140.50 |  |  |  |  |
|  | 10.00 | 168.99 |  |  |  |  |
|  | | | | | | |
| **Pumice**  **and Etch** | 1.00 | 165.12 | 142.72 | 165.72 | 151.23 | 8.72 |
|  | 2.00 | 155.53 |  |  |  |  |
|  | 3.00 | 145.97 |  |  |  |  |
|  | 4.00 | 155.09 |  |  |  |  |
|  | 5.00 | 142.90 |  |  |  |  |
|  | 6.00 | 144.28 |  |  |  |  |
|  | 7.00 | 142.72 |  |  |  |  |
|  | 8.00 | 146.48 |  |  |  |  |
|  | 9.00 | 148.52 |  |  |  |  |
|  | 10.00 | 165.72 |  |  |  |  |
